# Coping up with Stress as a Medical Student

**DOI:** 10.31729/jnma.7449

**Published:** 2022-04-30

**Authors:** Jiya Acharya, Shambhu Sahani

**Affiliations:** 1 Kathmandu Medical College and Teaching Hospital, Sinamangal, Kathmandu, Nepal; 2 KIST Medical College and Teaching Hospital, Imadol, Lalitpur, Nepal

**Keywords:** *burnout*, *expectations*, *medical student*, *psychological stress*

## Abstract

Psychological stress is one of the most common problems faced by medical students in their day to day life. There are a variety of manifestations of stress and these manifestations can directly or indirectly hamper the performance of a medical student. Knowing proper measures to manage stress as well as time can help medical students live their life in a more practical way. Practising mindfulness, mental health awareness and seeking help from experts can be beneficial in coping with stress as a medical student.

## INTRODUCTION

According to Hans Selye in 1936, "Stress is the nonspecific response of the body to any demand for change."^1^ It is one of the most common psychological problems faced by medical students and is present among 27% of the medical students.^2^ There are various factors that lead to stress in medical students including exams, the vastness of the subject, high family expectations, lack of time management, financial problems, relationship problems or even peer pressure.^3^ Due to this, stress is really a topic of discussion among medical students. Stress can manifest as a variety of symptoms that directly or indirectly hamper the normal day-to-day lifestyle of a medical student.

## AN EXPERIENCE

Well, being stressed is a vicious cycle in itself. Most medical students don't prefer seeking help for their own psychiatric problems, thus mostly present to mental health services once a crisis arises.^4^ Medical students are stressed as a result of their exams, which further leads to their inefficiency and decreased performance which ultimately makes it more arduous over time and this cycle continues on and on. Stress leads to problems like-altered bowel, palpitations, nervousness, emotional distress, anxiety, panic attack, triggering of pre-existing depression, easy fatigue, burn-outs, dryness of mouth, sleep disturbance, etc.^5^ When medical students are stressed, they cannot focus on the thing that they are doing, get annoyed very easily, become impatient, insomniac or even hypersomniac, feel sad and guilty over minor things and get tired easily. Emotional distress in medical students has been associated with substance abuse, broken relationships, suicidal ideation, thoughts of dropping out and on a more severe note, increased risk of brain drain and decreased productivity as a whole.^6^

Most of the medical students' days start with outnumbered thoughts in their minds. Fear of failure, the big syllabus which can't be finished in the given limited time are the biggest stress any medical student gets. Once failed, there would be a year of loss, and if it continues there is a chance of getting dropped out of medical school. In such a situation, one does not have the courage to face their peers who have passed, and in addition, have to adapt to a new environment with the juniors which is too difficult and eventually leads to them studying under pressure. Ultimately, they end up studying nothing and forgetting the things already studied. This often leads to them questioning their choices and decisions. All these instances lead to low self-esteem and confidence. On top of this, fear of ragging is another completely neglected part of medical school. There have been situations where medical students have killed themselves due to extreme levels of torture and psychological fear of ragging. Thus, the amount of mental pressure a student has to bear throughout medical school can never be imagined. And in that phase, most medical students are confused about what step to take. Extremely high family expectations and lack of support from family can as well lead to stress and anxiety in most students which bring out changes in the serum level of many hormones including glucocorticoids, catecholamines, growth hormones and prolactin and these chemical changes are necessary for the fight or flight response of the body as an adaptation to stress.^7^

## OVERCOMING THE STRESS

Medical exams can be nerve-racking most of the time. But knowing the proper ways to handle the stress and frustration during exams can make it more smooth in the long run. Staying at hostels, away from home and family, can make the situation more challenging. So, talking to the family during exams even for a few minutes can be more calming and assuring at the same time. After exams, a short nap can better help to prepare for the next one. Also, spaced repetitions can help to retain things with more ease. Some of the medical students get used to the stress and know how to handle it. However, some end up with mental stress and mental illnesses.

Knowledge of stress and how to manage it is a must for medical students both as a part of their study and also as part of managing their life. As a medical student, it is equally important to take care of your health besides patients'. Stress not only impacts our health and personal growth but also can also influence the way we deal with patients or even answer during our viva. Most medical students are unable to properly manage their time and hence, fail to multitask; resulting in piling up of work and stressful last moment. As a result, they become unable to take a good rest and have a good sleep. 44.23% of medical students have poor sleep quality which is more prevalent among female students.^8^

To maintain effectiveness in our day-to-day life, we need to take proper care of our sleep and sleep patterns. Taking at least 6-8 hours of continuous sleep without any disturbances is an excellent way to increase efficiency. Learning yoga, meditation, and doing regular aerobic exercises can be effective in helping with stress management. These activities tend to release endorphins from the brain that makes us feel good and distract us from worries. They also improve mood and help in better command over our body and life. Apart from these, practicing breathing exercises can help one to stay calm. As a medical student, these moments of stress aren't something that one can avoid or stop totally, so having a good command over the way we handle situations can be a good start to seeking balance in life.

## WAY FORWARD

All in all, a significant number of medical students suffer from mental illness every year, mostly due to the stress they perceive during their med-school life. Stress has led to suicidal thoughts, college dropout, severe psychosis, decreased performance and even inability to balance professional and personal life. In the course of sacrifices and commitments, medical students tend to be stressed not only from their studies but also from the high hopes of family and society. Normalizing medical students seeking help can bring remarkable changes in the life of medical students and medical professionals as a whole. Mental health awareness, practicing breathing exercises, friends and family support, seeking help from experts, and proper time management can be effective ways to overcome stress.
